# Lessons learned from the COVID-19 control strategy of the XXXII Tokyo Summer Olympics and the XXIV Beijing Winter Olympics

**DOI:** 10.1080/22221751.2022.2090859

**Published:** 2022-07-04

**Authors:** Yi Zhang, Jingwen Ai, Jing Bao, Wenhong Zhang

**Affiliations:** a Department of Infectious Diseases, National Medical Center for Infectious Diseases, Shanghai Key Laboratory of Infectious Diseases and Biosafety Emergency Response, Huashan Hospital, Fudan University, Shanghai, People’s Republic of China; b Shanghai HealthCare Capital, Shanghai, People’s Republic of China; c State Key Laboratory of Genetic Engineering and Institute of Biostatistics, School of Life Sciences, Fudan University, Shanghai, People’s Republic of China

**Keywords:** COVID-19, Olympics, prevention, control, strategy

## Abstract

Coronavirus disease 2019 (COVID-19) has caused more than 500 million infections and 6.2 million deaths globally, resulting in numerous sporting events being cancelled or postponed. Therefore, effective control strategies are urgently needed to prevent COVID-19 transmission at these local and global events. This article introduced the strategies utilized at the Tokyo and Beijing Olympics and proposed several measures for future reference.

There have been more than 500 million COVID-19 infections across the globe through April 2022 [1]. The virus has caused widespread disruptions in the health care, economic, and educational systems. In efforts to prevent further spread of the virus, approximately 500 social and recreational events were cancelled or delayed [2]. Non-pharmaceutical interventions (NPIs) have proven to be an important strategy to control this pandemic, including lockdowns, school closures, mask wearing, and social distancing [3,4]. However, these measures may negatively impact economic, educational, and social activities and are therefore heavily reliant on community acceptance [5]. Vaccination has prevented the virus transmission to a certain degree; however, breakthrough infections and emergent new variants have worsened the COVID-19 epidemic [6]. Thus far, most countries have issued their own policies in response to the COVID-19 pandemic. While the international sporting events help to connect the world through this difficult period, a number of challenges have posed to the host cities, their citizens, and participating athletics from around the world. Specific COVID-19 prevention and control strategies, including NPI and vaccinations, are needed to reduce the risks of COVID-19 transmission during these international sporting events.

## Challenges and potential risks for hosting the international sporting events under the COVID-19 epidemic

Several outbreaks in previous mass-sporting events [7] have emphasized that the COVID-19 and other infectious disease outbreaks might expose athletes at risk. The COVID-19 risk factors include crowd density, duration of the events, method of transportation, distance between individual members of the audients, distance between athletic participants, and the ongoing changing status of COVID-19 pandemic [8].

Several public health issues should also be considered, including whether additional precautions should be taken for unvaccinated people, Paralympic athletes, and immunocompromised people who may be at higher risk for infection. During the events, athletes, team staff, media personnel, and others, maybe in close contact with one another which may further increase the risk of infection. Even if the strictest measures are taken, sporadic outbreaks of infections may still occur.

## Experience from Tokyo and Beijing Olympics regarding COVID-19 prevention and control strategies

The Tokyo Olympic Committee and the Beijing Olympics Committee each introduced several strategies to reduce COVID-19 transmission during the Olympic and Paralympic Games [9–12]. The Tokyo Olympic Committee recommended four main principles: Mask wearing; COVID-19 test, trace, and isolate; Minimizing physical interaction; and Think hygiene. The Beijing Olympic Committee utilized the following strategies: Vaccination; Closed loop; COVID-19 Liaison Officers; Test, trace and isolate; Minimise physical interaction and Think hygiene (Table 1**)**.

**Table 1.** COVID-19 control strategies in Tokyo Olympics and Beijing Olympics.

Principles	Measures in Tokyo Olympics	Measures in Beijing Olympics	Differences
Vaccination	Although vaccination was not mandatory, 85% vaccination coverage was finally achieved among resident in Olympics village.	It is mandatory to be fully vaccinated at least 14 days prior to departure for China, in order to be allowed in the closed loop system without quarantine. Anyone not fully vaccinated needs to be quarantine for 21 days upon arrival in Beijing.	Beijing Olympics required participants to be fully vaccinated at least 14 days prior to departure for China while Tokyo Olympics did not require vaccination.
Closed Loop	Absence of strict closed-loop system, but a bubble system was adopted. In the isolation bubble system, athletes were restricted to Games venues and limited to additional locations. They used transportation dedicated for the Olympics, and could only spend time with people on a pre-submitted list of contacts.	Participants were subjected to daily health monitoring for all games and testing within the closed loop, and will be allowed to travel between the designated sites in dedicated Olympic transportation.	Both Olympics implied closed-loop system and restricted the activities of the participants.
Mask wearing	Mask wearing at all times except when eating, drinking or sleeping.	Though not listed as one of the principles, mask wearing was included in the “Think hygiene” principle.	“Think hygiene” principle was applied to Beijing Olympics beside mask wearing.
Test, trace and isolate	**Test** **(1) Testing before departure** Two COVID-19 tests on two separate days within 96 h before departure to Japan **(2) Testing again upon arrival at the airport** **(3) Regular testing** Athletes and officials: Have regular screening tests for COVID-19 daily (Initial test: saliva antigen test; if positive: saliva PCR test) The other six populations (International Federations; press; broadcasters; Olympic and Paralympic families; marketing partners and workforce): daily test within the first three days, and accept testing daily, every four days or every seven days depending on the frequency of contact, and each must be tested 14 days after arrival.	**Test** **(1) Testing before departure** Two COVID-19 tests on two separate days within 96 h before departure to China. **(2) Testing again upon arrival at the airport** **(3) Daily PCR screening tests for COVID-19** **Trace** Download the “My 2022” application to monitor and track health dally for 14 days before departure for China **Isolate** Anyone not fully vaccinated but eligible to travel to China based on the criteria above must be quarantined for 21 days upon arrival	(1) COVID-19 test frequency: In Tokyo Olympics, athletes and officials had regular screening tests for COVID-19 daily; the other populations received daily tests within the first three days, and accept testing daily, every four days or every seven days depending on the frequency of contact. Daily PCR screening tests for COVID-19 in Beijing Olympics. (2) Trace: different applications were used in the two Olympics (3) Isolate: Participants not fully vaccinated were quarantined for 21 days upon arrival while for the first three days in Tokyo Olympics;
	**Trace** Contact confirming application (COCOA)		
	**Isolate** On arrival and the next three days but have permission to perform activities if test negative for COVID-19 every day; and operate under a higher level of supervision.		
Minimum physical interaction	Follow “Activity Plan” Limit contact with other people Keep a distance of two metres Avoid physical contact, hugs, high-fives and handshakes Avoid enclosed spaces and crowds Use dedicated Games vehicles	Keep physical distance Avoid physical contact Keep two metres’ distance from the athletes and at least one metre from others Avoid enclosed spaces and crowds Use dedicated Games transport. Only carry out the activities relevant for role at the Games	/ No obvious differences
Think hygiene	Wear a face mask at all times Wash hands regularly and used hand sanitizer where available Support athletes by clapping instead of singing or chanting Avoid using shared items where possible, or disinfect them Ventilate rooms and common spaces every 30 min	Properly wear a face mask at all times (KN95, N95, FFP2, or equivalent standard of protection) Wash hands regularly and used hand sanitizer where available Support athletes by clapping instead of singing or chanting Avoid using shared items where possible, or disinfect them Ventilate Regularly the rooms you are staying	/ No obvious differences
COVID-19 Liaison Officers (CLOs)	Though not listed as one of the principles, CLOs engaged the whole Games.	Every organization taking part in the Games has nominated their COVID-19 Liaison Officers. CLOs support and help sports participants.	/ No obvious differences

For all athletic participants of Beijing Olympics, it was mandatory to be fully vaccinated at least 14 days prior to arriving in China in order to be allowed in the closed loop system without quarantine. The unvaccinated participants were quarantined for 21 days upon arrival in Beijing. Although vaccination was not mandatory at the Tokyo Games, 85% of the residents in Tokyo Olympics Village were vaccinated [13].

The game-time closed-loop management system was employed in Beijing Olympics to keep the participants and the local people safe through reducing unnecessary interactions. This system allowed the participants to enter China without a 21-day quarantine if fully vaccinated. Participants followed daily health monitoring, testing, and used the dedicated commute between the permitted destinations (including Games venues, accommodation facilities, etc.), bus, taxi or train reserved for Olympics purposes. In the Tokyo Olympics, though a strict closed-loop system was absent, a bubble system was adopted [14]. In the isolation of the bubble system, athletes were restricted to Games venues and limited additional locations. They used transportation dedicated for the Olympics, and could only interact with people on a pre-submitted list of contacts.

During Tokyo Olympics, regular saliva screening tests for COVID-19 were performed. Participants were isolated upon arrival for three days and each participant had to complete a detailed form listing all the recent contacts. The Beijing Olympics performed daily polymerase chain reaction (PCR) screening tests using the oropharyngeal (throat) swabs for all participants. Application “My 2022” was used to monitor and track daily health status for 14 days before departing for China.

Social distancing measures during the Tokyo Olympics included limiting individual contacts and keeping a two-metres distance between the individuals. Technologies such as smart disinfection machines and cooking robots had played an important role in Beijing Olympics. The numbers of spectators were restricted for both Olympics, approximately 43.3 thousand for Tokyo Olympic and 110 thousand for Beijing Olympic [15,16]. “Think Hygiene” focused on washing hands, not sharing personal items, and adequate room ventilations. Though not listed as one of the principles, mask wearing was included in the “Think Hygiene” principle. Beijing Olympics recommended KN95, N95, FFP2 masks, or the equivalent standards of protection to effectively protect Olympic participants [11,12].

Both Tokyo and Beijing Olympics COVID-19 control strategies were effective in limiting the transmission of COVID-19. From 1st July to 8th August, approximately 430 COVID-19 infections were identified at the Tokyo Olympics [17,18]. Of the 430 confirmed cases at the Tokyo Olympics, contract workers represented the majority (54.88%, 236/430), followed by 109 (25.35%) other Games participants (including personnel, volunteer, employee and media) and 29 (6.74%) athletes. The Beijing Olympics Committee confirmed 437 COVID-19 infections from 23rd January to 20th February. Among Beijing Olympics COVID-19 cases, 265 positive cases were detected upon arrival at the airport, including 116 (43.77%) athletes and team officials, and 149 (56.23%) other participants. The remaining 172 cases were diagnosed in the “closed-loop” system, encompassing 69 athletes and team officials, and 103 other participants [19, 20]. Therefore, more strict prevention and control measures could be applied for the nonathletes in the future events.

**Figure 1.**
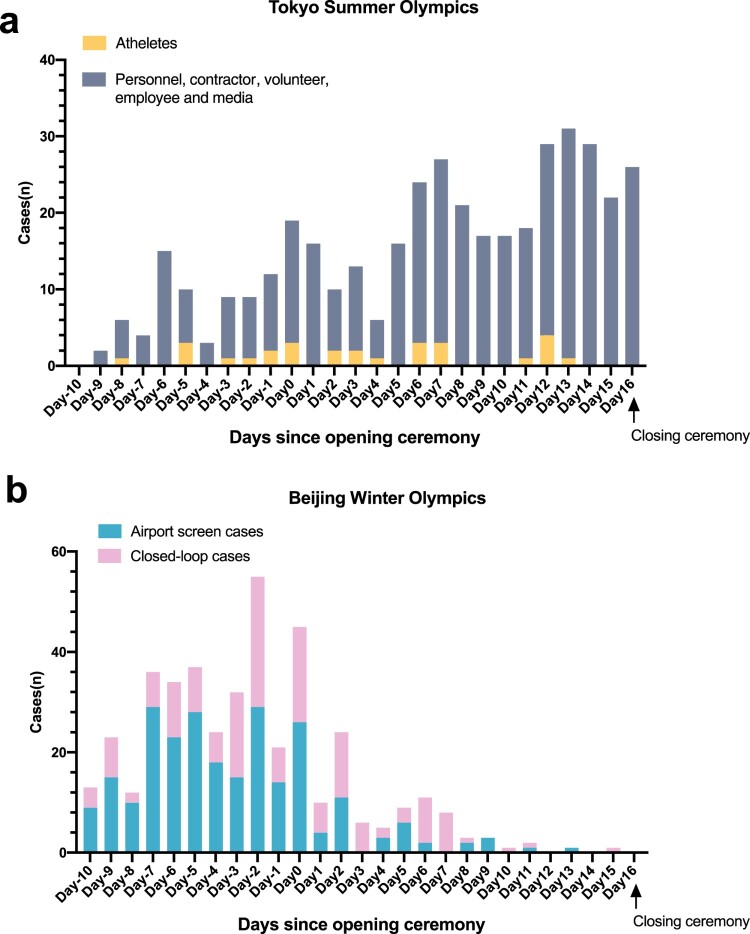
The dynamics of COVID-19 detection during Olympics. (a) The dynamics of COVID-19 detection in Tokyo Summer Olympics. The yellow and grey bars represented the cases of athletes and the other attendees including contractors, volunteers, employee and media personnel, respectively. (b) The dynamics of COVID-19 detected cases and proportion in Beijing Winter Olympics. The blue and pink bar showed the cases from airport screen and closed-loop.

The COVID-19 case detection dynamics during the two Olympics are further illustrated in Figure 1 [19,20]. Due to the foreign participants entering the host country, there was an increase in COVID-19 cases 10 days before the opening ceremony in both Olympics After the opening ceremony, the number of COVID-19 cases in Tokyo continued to rise. However, in the Beijing Winter Olympics, the number of cases dropped dramatically after entering Beijing's stringent “closed-loop” system. The Tokyo Olympics had a larger scale than the Beijing Olympics, with more sporting events and participants. The number of athletes at the Tokyo and Beijing Olympics were 11,420 and 2834, respectively [21,22], and therefore there was a higher potential risk of transmission at the Tokyo Olympics. The Delta variants were most prevalent at the Tokyo Olympics in 2021 while the Omicron was the dominant variant at the Beijing Olympics in the winter of 2022. The Omicron variant had an incubation period of 2.9 days compared to 3.2 days for the Delta variant. The Omicron variant also had stronger transmissibility, and lower neutralization sensitivity [23–26]. Furthermore, the Tokyo Olympics took place in the summer when COVID-19 transmission is generally expected to be lower [27].

## Recommended COVID-19 prevention and control strategies in the future

We propose several measures to decrease COVID-19 transmission rates at future Olympics and other major sporting events based on the experiences from the Tokyo and Beijing Olympics. The bubble system and the closed-loop system are both highly effective prevention strategies. New approaches could be adopted in the future depending on the real-time cases and risk analysis of the host city. The combination of frequent testing vaccinations, social distancing, and mask mandate during the competition will help decrease the COVID-19 transmission rate. These prevention measures may be modified as the new vaccines and the effective medications become available.

Organizers of future sporting events need to frequently remind attendees that although preventive measures such as mask wearing, social distancing, and personal hygiene are somewhat inconvenient, they have proven to be highly effective at reducing transmission of COVID-19 [28]. Furthermore, more testing centres should be arranged in the event areas and all participants will be encouraged to receive vaccinations prior to arrival. More medical service centres and emergency response plans for the competition should be developed and emergency response plans should anticipate the possibility of sporadic increases in the number of cases. Additionally, the COVID-19 infection of the host countries and cities should be communicated in real-time to further guide the prevention practice.

Olympic Games are one of the few events that can truly connect people on the global level. The spirit and benefits the Olympic Games bring to the world is hugely encouraging and influential. The lessons learned from the two Games, Beijing Winter Olympics and Tokyo Summer Olympics, are invaluable for the future sporting events as well as other international gatherings. By adopting the proven technologies and measures, it is possible to hold relatively safe and successful international events in the future. The two Olympics also provided us with treasured experience in bridging international differences in epidemic prevention and control. The strategy can be extended to political, economic, and academic communications to promote global interaction and understanding before and during the global epidemic.
